# Derivation of Bose’s Entropy Spectral Density from the Multiplicity of Energy Eigenvalues

**DOI:** 10.3390/e26060504

**Published:** 2024-06-09

**Authors:** Arnaldo Spalvieri

**Affiliations:** Dipartimento di Elettronica, Informazione e Bioingegneria, Politecnico di Milano, 20133 Milan, Italy; arnaldo.spalvieri@polimi.it

**Keywords:** blackbody radiation, Bose–Einstein distribution, degeneracy of energy eigenvalues, multiplicity of energy eigenvalues, density of states

## Abstract

The modern textbook analysis of the thermal state of photons inside a three-dimensional reflective cavity is based on the three quantum numbers that characterize photon’s energy eigenvalues coming out when the boundary conditions are imposed. The crucial passage from the quantum numbers to the continuous frequency is operated by introducing a three-dimensional continuous version of the three discrete quantum numbers, which leads to the energy spectral density and to the entropy spectral density. This standard analysis obscures the role of the multiplicity of energy eigenvalues associated to the same eigenfrequency. In this paper we review the past derivations of Bose’s entropy spectral density and present a new analysis of energy spectral density and entropy spectral density based on the multiplicity of energy eigenvalues. Our analysis explicitly defines the eigenfrequency distribution of energy and entropy and uses it as a starting point for the passage from the discrete eigenfrequencies to the continuous frequency.

## 1. Introduction

The most important step after the fundamental paper [1] of Planck on the energy spectral density of the blackbody radiation was moved by Bose in [2], whose result was immediately extended by Einstein in [3] to massive particles, leading to the Bose–Einstein distribution of energy. Entropy plays a key role both in Planck’s derivation and in Bose’s derivation of the energy spectral density of the radiation. Today, the entropy calculated by Bose is recognized to be the equal to the thermodynamic entropy of the blackbody radiation.

Bose’s derivation is based on two ingredients. One is the geometric probability distribution of the number of energy quanta that occupy one quantum state, the other is the so-called *density of states*, that is, the infinitesimal number of quantum states in an infinitesimal interval of frequency. Bose’s calculation of the density of states is based on the quantization of the phase space into cells of volume $h^{3}$, where *h* is Planck’s constant. This approach, where one cell is a quantum state, is today surpassed by the modern quantum approach, where the quantum states used for the calculation of the density of states are the quantum eigenstates obtained by imposing the boundary conditions determined by the spatial boundaries of the body.

In the case of massive particles, the relationship between multiplicity of energy eigenvalues and density of states is well known and widely discussed, see for instance [4] and 1.4 of [5]. However, in the case of photons, today’s textbook treatment completely skips the multiplicity of energy eigenvalues in the derivation of the density of states and, with it, it skips the discrete energy spectrum at the eigenfrequencies corresponding to the energy eigenvalues.

In this paper we fill this gap. First we derive the discrete spectrum of energy and entropy at system’s eigenfrequencies, then we pass from the discrete to the continuous frequency, finding in this way Planck’s energy distribution and Bose’s entropy distribution.

The outline of the paper is as follows. In Section 2, we discuss the relationship between the multiplicity of energy eigenvalues and the density of states in the continuous frequency domain for photonic systems. Section 3 introduces the discrete spectrum of energy and entropy and shows that the passage to the continuous frequency leads to Planck’s energy spectral density and Bose’s entropy spectral density. Section 4 compares our derivation to other derivations. Section 5 discusses the difference between the entropy of the thermal state and that of a set of harmonic oscillators at the coherent state with the same energy distribution, showing that the difference between the two is really small. Finally, in Section 6 we draw the conclusions.

## 2. Multiplicity of Energy Eigenvalues and Density of States

Consider photons inside a cubic reflective cavity of side *L*. By imposing the boundary conditions, one finds by standard arguments that the energy eigenvalues are

$$\epsilon_{i,j,k}=h\nu_{i,j,k}=\frac{hc}{2L}\sqrt{i^{2}+j^{2}+k^{2}},\;\;\left(i,j,k\right)\in N^{3}\setminus \left(0,0,0\right),$$

where the triple of natural numbers (i,j,k) identifies the eigenstate of the photon in the considered polarization, $\nu_{i,j,k}$ is the eigenfrequency (i,j,k), *c* is the speed of the light and *h* is the universal constant that Planck determined in [1] to be equal to $6.55\cdot 10^{-34}$ J· s, today $h=6.62\cdot 10^{-34}$ J· s, by imposing compatibility between his theory and the experimental evidence. Let $r_{i,j,k}$ be the random occupancy number of eigenstate (i,j,k), i.e., the random number of photons whose eigenstate is (i,j,k). The energy and the frequency of the photon depend not on the specific triple (i,j,k) but only on the sum of their squares, that is, only on the following integer *n*:

(1) $$n=i^{2}+j^{2}+k^{2},$$

(2) $$\epsilon_{n}=h\nu_{n}=\frac{hc}{2L}\sqrt{n}.$$

Let $M_{n}$ be the set of triples (i,j,k) that are distinct roots of (1) and let $m_{n}$ be the multiplicity, or degeneracy, of $\epsilon_{n}$, that is the number of triples in $M_{n}$. For instance,

$$M_{2}=\left\{\left(0,1,1\right);\left(1,0,1\right);\left(1,1,0\right)\right\},\;m_{2}=3,$$

$$M_{7}=\emptyset ,\;m_{7}=0.$$

The first values of $m_{n}$ are $m_{1}=m_{2}=m_{4}=m_{6}=m_{8}=3$, $m_{3}=1$, $m_{5}=m_{9}=6$ (with $9=1^{2}+2^{2}+2^{2}=3^{2}+0^{2}+0^{2}$), $m_{7}=0$. Then the sequence proceeds with wild irregularities. Other examples of $m_{n}$ for n>9 are $m_{38}=9$ (with $38=1^{2}+1^{2}+6^{2}=2^{2}+3^{2}+5^{2}$), $m_{39}=0$, $m_{40}=6$, $m_{62}=12$ (with $62=1^{2}+5^{2}+6^{2}=2^{2}+3^{2}+7^{2}$), $m_{63}=0$. By Legendre’s three squares theorem, all the natural numbers excepting those of the form $n=4^{a}\left(8b+7\right)$, $\left(a,b\right)\in N^{2}$, can be represented as the sum of three squares, therefore $m_{4^{a}\left(8b+7\right)}=0$. Also, for convenience in the following we will put $m_{0}=0$.

The relationship between the multiplicity of energy eigenvalues and the density of states is well known and widely discussed in the context of massive particles, see, e.g., [4]. These arguments are based on the observation that the number of the points (eigenstates) with non-negative integer coordinates (i,j,k) that satisfy the inequality

$$n=i^{2}+j^{2}+k^{2}\leq N$$

is equal to the number of points contained in the positive octant of a sphere of radius $\sqrt{N}$. By quantizing the three-dimensional space into cubic cells of the unit side, hence of unit volume, we recognize that the number of points (eigenstates) that satisfy the above inequality is approximated to the volume of the positive octant of the sphere of radius $\sqrt{N}$:

(3) $$\begin{array}{c} \sum_{n=0}^{N}m_{n}\approx \int_{s=0}^{N}\frac{\pi }{4}\sqrt{s}ds. \end{array}$$

Actually, since

$$\int_{s=0}^{N}\frac{\pi }{4}\sqrt{s}ds=\frac{\pi }{6}\sqrt{N^{3}},$$

the right hand side of (3) is just the mentioned volume. By uniformly sampling the argument of the integral with unit step, we have the approximation

(4) $$\begin{array}{c} \sum_{n=0}^{N}m_{n}\approx \sum_{n=0}^{N}\frac{\pi }{4}\sqrt{n}, \end{array}$$

which suggests that we should regard the argument of the sum in the right hand side as a smooth but still discrete version of the true multiplicity. For N→∞, the relative error in the approximation (3) becomes vanishingly small, leading to

(5) $$\begin{array}{c} \lim_{N\to \infty }\frac{4\sum_{n=0}^{N}m_{n}}{\pi \int_{0}^{N}\sqrt{s}ds}=1. \end{array}$$

The above limit is deeply discussed in 1.4 of [5] in the context of the analysis of multiplicity of energy eigenvalues of massive particles.

The arguments discussed till here can be used both in the analysis of multiplicity of energy eigevalues of systems of massive particles and of systems of photons. The difference between the two cases comes out when we consider Equation (2), which is specific for photons and leads us to define the continuous frequency ν as

(6) $$\nu =\frac{c}{2L}\sqrt{s},\;\;\;V=\frac{c}{2L}\sqrt{N},$$

where V is the eigenfrequency that maps on the surface of the sphere. Looking at the integral in (3) as at a sum between n=0 and n=N, we regard the integration variable *s* as the continuous version of index *n*, therefore, after the change of variables from *s* to ν in the integral (3), we obtain

(7) $$\begin{array}{c} \sum_{n=0}^{N}m_{n}\approx \int_{\nu =0}^{V}\frac{4\pi L^{3}\nu^{2}}{c^{3}}d\nu . \end{array}$$

The fraction appearing inside the integral is called the *density of states* per polarization, which, multiplied by dν, is interpreted as the infinitesimal number of quantum states between frequency ν and frequency ν+dν per polarization.

The above density of states holds also for cavities with other geometries than the cube, provided that the volume of the cavity is equal to the volume $L^{3}$ of the cubic cavity, see [4] for the case of massive particles. For instance, when the cavity is a rectangular parallelepiped with sides $L_{x},L_{y},L_{z},$ the eigenfrequecies are

$$\nu_{i,j,k}=\frac{c}{2\sqrt[{3}]{V_{cavity}}}\sqrt{x_{i}^{2}+y_{j}^{2}+z_{k}^{2}},\;\;\left(i,j,k\right)\in N^{3}\setminus \left(0,0,0\right),$$

with $V_{cavity}=L_{x}L_{y}L_{z}$ and

$$x_{i}=\frac{i\sqrt[{3}]{V_{cavity}}}{L_{x}},\;y_{j}=\frac{j\sqrt[{3}]{V_{cavity}}}{L_{y}},\;z_{k}=\frac{k\sqrt[{3}]{V_{cavity}}}{L_{z}}.$$

If $L_{x}$, $L_{y}$, $L_{z},$ are non-commensurable between them, then the multiplicity of all the eigenfrequencies is one and the number of eigenfrequencies such that

$$\nu_{i,j,k}\leq V$$

is equal to the number of points with integer coordinates (i,j,k) contained in the first octant of the ellipsoid with semi-axes

$$A_{x}=\frac{2VL_{x}}{c},\;A_{y}=\frac{2VL_{y}}{c},\;A_{z}=\frac{2VL_{z}}{c}.$$

The mentioned number of points is approximated to the volume of the first octant of the ellipsoid, which, expressed in spherical coordinates is,

$$\begin{array}{cc} V_{ellipsoid} & =\int_{\theta =0}^{\pi /2}\int_{\phi =0}^{\pi /2}\int_{\rho =0}^{1}\rho^{2}A_{x}A_{y}A_{z}\sin\left(\phi \right)d\theta d\phi d\rho \\ & =\frac{\pi }{2}\int_{\rho =0}^{1}\rho^{2}A_{x}A_{y}A_{z}d\rho \\ & =\int_{\nu =0}^{V}\frac{4\pi V_{cavity}\nu^{2}}{c^{3}}d\nu , \end{array}$$

where, in the last equality, we substitute ν=ρV.

## 3. Energy Spectral Density and Entropy Spectral Density of the Photonic Thermal State

Consider the thermal state and let $r_{n}$ be the random number of photons that occupy a quantum eigenstate whose three quantum numbers belong to $M_{n}$. By standard maximization of Shannon entropy with constrained mean value, one finds that, whichever is the specific triple $\left(i,j,k\right)\in M_{n}$, the probability distribution of the random occupancy number is geometric:

$$P_{r_{n}}=\left(1-e^{-\beta h\nu_{n}}\right)e^{-r\beta h\nu_{n}},\;\;r_{n}=0,1,\ldots ,$$

where $\beta ={\left(k_{B}T\right)}^{-1}$ is the inverse temperature, $k_{B}=1.38\cdot 10^{-23}$ J/K is the Boltzmann constant, *T* is the temperature in Kelvin degrees and ϵ is the energy of the photon. The mean value of the random variable $r_{n}$ is the Bose statistics

$$\mu_{r_{n}}=\frac{1}{e^{\beta h\nu_{n}}-1}.$$

Probability distribution, variance and entropy can be expressed as a function of the mean value:

$$P_{r_{n}}=\frac{1}{1+\mu_{r_{n}}}{\left(\frac{\mu_{r_{n}}}{1+\mu_{r_{n}}}\right)}^{r_{n}},$$

$$\sigma_{r_{n}}^{2}=\mu_{r_{n}}\left(\mu_{r_{n}}+1\right),$$

(8) $$\begin{array}{cc} S_{r_{n}} & =\log\left(1+\mu_{r_{n}}\right)+\mu_{r_{n}}\log\left(1+\mu_{r_{n}}^{-1}\right)\\ & =\frac{\beta h\nu_{n}}{e^{\beta h\nu_{n}}-1}-\log\left(1-e^{-\beta h\nu_{n}}\right), \end{array}$$

where the entropy is expressed in $k_{B}$ units and the first term in the last line is β times the expectation of the sum of the energies of the random number of photons that populate the quantum state at hand. It is easy to see that

(9) $$S_{r_{n}}\geq \mu_{r_{n}}\left(1-\log\left(\mu_{r_{n}}\right)\right),$$

which holds with equality for $\mu_{r_{n}}\to 0$.

Assuming independency between the occupancy numbers, the joint probability distribution of vector $\bar{r}$ of the occupancy numbers is

$$P_{\bar{r}}=\prod_{n=0}^{\infty }P_{r_{n}}^{m_{n}}.$$

**Figure 1:**
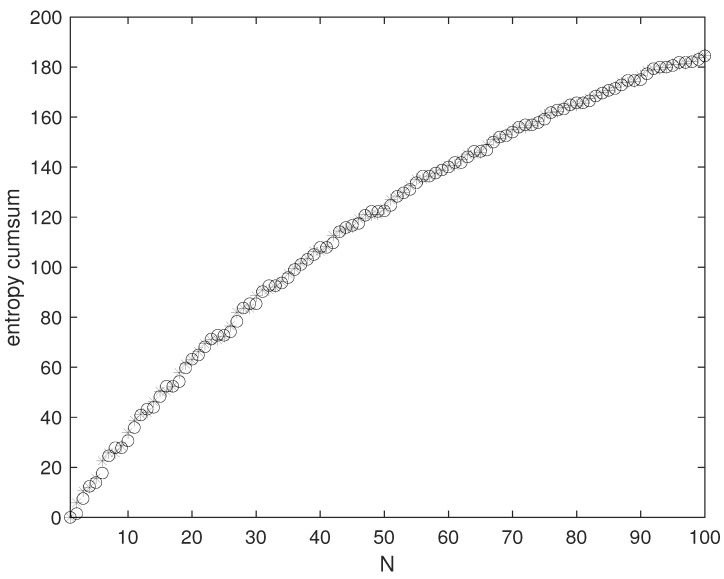
Sum (11) versus *N*. Temperature 300 Kelvin, side of the box $6\cdot 10^{-5}$ m. Asterisks: exact entropy based on the true multiplicity $g_{n}=m_{n}$. Circles: approximation obtained using the smooth multiplicity $g_{n}=\pi \sqrt{n}/4$.

Due to independency of the individual occupancy numbers, the total entropy of the considered polarization is the sum of the entropies of the occupancy numbers of the individual eigenstates

(10) $$\begin{array}{cc} S_{\bar{r}} & =\sum_{n=0}^{\infty }m_{n}S_{r_{n}}\\ & =\sum_{n=0}^{\infty }\frac{m_{n}\beta h\nu_{n}}{e^{\beta h\nu_{n}}-1}-m_{n}\log\left(1-e^{-\beta h\nu_{n}}\right), \end{array}$$

where the first term in the second line is β times the energy distribution in the eigenfrequencies. Figure 1 reports the cumulative sum

(11) $$\begin{array}{c} \sum_{n=0}^{N}g_{n}S_{r_{n}}, \end{array}$$

with the true multiplicity $g_{n}=m_{n}$ and with the smooth multiplicity $g_{n}=\pi \sqrt{n}/4$, see the right hand side of (4).

Defining the entropy spectral density as the continuous version of the entropy distribution with smooth multiplicity $S_{r_{n}}\pi \sqrt{n}/4$, hence as the product between the density of states and the entropy of the geometric distribution expressed as a function of the continuous frequency, that is

(12) $$\mathrm{entropy}\;\mathrm{spectral}\;\mathrm{density}\overset{\mathrm{def}}{=}\frac{4\pi L^{3}\nu^{2}}{c^{3}}S_{r}\left(\nu \right),$$

where, from (8),

$$S_{r}\left(\nu \right)=\frac{\beta h\nu }{e^{\beta h\nu }-1}-\log\left(1-e^{-\beta h\nu }\right),$$

and integrating it, we arrive at Bose’s entropy per polarization. Note that, due to an error, the sign of the exponent in the exponential in the second-last equation of [2] is flipped.

(13) $$\begin{array}{cc} S_{\bar{r}} & \approx \int_{\nu =0}^{\infty }\frac{4\pi L^{3}\nu^{2}}{c^{3}}\left(\frac{\beta h\nu }{e^{\beta h\nu }-1}-\log\left(1-e^{-\beta h\nu }\right)\right)d\nu \\ & =\frac{16\pi^{5}L^{3}}{45\beta^{3}h^{3}c^{3}}=108.8\cdot \frac{L^{3}}{\beta^{3}h^{3}c^{3}}, \end{array}$$

where the first addend inside the integral is β times Planck’s energy spectral density per polarization. The calculus of the integral is standard. Specifically, it is based on

$$\int_{x=0}^{\infty }\frac{x^{3}}{e^{x}-1}dx=\frac{\pi^{4}}{15},\;\;-\int_{x=0}^{\infty }x^{2}\log\left(1-e^{-x}\right)dx=\frac{\pi^{4}}{45}.$$

## 4. Comparison with Other Derivations

Bose’s approach is semi-classical. He computes the infinitesimal number of quantum states that a photon with frequency between ν and ν+dν can occupy as the infinitesimal number of cells of volume $h^{3}$ contained in the region of the six-dimensional phase space of the photon where the sum of photon’s three squared momenta is between ${\left(h\nu /c\right)}^{2}$ and ${\left(h\left(\nu +d\nu \right)/c\right)}^{2}$. The volume of the mentioned region in the three spatial coordinates is $L^{3}$, while in the domain of the three momenta $\left(p_{x},p_{y},p_{z}\right)$ the infinitesimal volume is that of a spherical shell between radia hν/c and h(ν+dν)/c, therefore the sought infinitesimal number of cells is

(14) $$\begin{array}{cc} \frac{L^{3}}{h^{3}}\int_{\sqrt{p_{x}^{2}+p_{y}^{2}+p_{z}^{2}}=h\nu /c}^{h(\nu +d\nu )/c}dp_{x}dp_{y}dp_{z} & =\int_{u=\nu }^{\nu +d\nu }\frac{L^{3}4\pi u^{2}}{c^{3}}du\\ & =\frac{L^{3}4\pi \nu^{2}}{c^{3}}d\nu , \end{array}$$

where the first equality is based on the change of variables from Cartesian coordinates to spherical coordinates with radial coordinate hu/c:

(15) $$\sqrt{p_{x}^{2}+p_{y}^{2}+p_{z}^{2}}=\frac{h}{c}u,\;\;dp_{x}dp_{y}dp_{z}=\frac{h^{3}4\pi u^{2}}{c^{3}}du,$$

and (14) is just the argument of the integral (7).

Pathria, in chapter 6 of [5], completely skips the discrete domain and basically follows the same approach of Bose, excepting that Pathria refers to (14) as to the *Rayleigh expression* for the (infinitesimal, we add) *number of normal modes of vibration*.

The standard modern approach of, among others [6,7,8,9,10], starts from the three quantum numbers (i,j,k) and derives the energy spectral density (the entropy spectral density can be derived in a completely similar manner) by considering a continuous triple (x,y,z) in place of (i,j,k):

(16) $$\begin{array}{cc} \; & \sum_{i,j,k}\frac{h\nu_{i,j,k}}{e^{\beta h\nu_{i,j,k}}-1}=\sum_{i,j,k}\frac{hc\sqrt{i^{2}+j^{2}+k^{2}}}{2L(e^{\beta hc\sqrt{i^{2}+j^{2}+k^{2}}/2L}-1)}\\ & \approx \int_{x,y,z=0}^{\infty }\frac{hc\sqrt{x^{2}+y^{2}+z^{2}}}{2L(e^{\beta hc\sqrt{x^{2}+y^{2}+z^{2}}/2L}-1)}dxdydz \end{array}$$

(17) $$\begin{array}{cc} & =\frac{1}{8}{\left(\frac{2L}{c}\right)}^{3}\int_{\nu =0}^{\infty }\frac{h\nu }{e^{\beta h\nu }-1}4\pi \nu^{2}d\nu , \end{array}$$

In the above sequence of passages, the last equality is obtained by changing the integration variables from Cartesian coordinates to spherical coordinates, that is, putting

(18) $$\sqrt{x^{2}+y^{2}+z^{2}}=\frac{2L}{c}\nu ,\;\;dxdydz={\left(\frac{2L}{c}\right)}^{3}4\pi \nu^{2}d\nu ,$$

where the radial coordinate 2Lν/c is the continuous version of our $\sqrt{n}$. Since now only non-negative values of (x,y,z) are allowed, the factor 1/8 in front of the integral restricts the domain of the integral to the positive octant of the three-dimensional space spanned by (x,y,z). A very similar derivation can be found in 52 of [11]. An exception is Feynman, who derives the energy spectrum in an inherently continuous manner that is neither based on the discretization of phase space nor on the imposition of the boundary conditions, see 41-2 and 41-3 of [12]. However, Feynman discusses only the expected energy, not the entire probability distribution, so he does not derive the entropy. The approach of Feynman seems to better fit the dense nature of the energy spectrum obtained from the measurements, while the approach based on the imposition of the boundary conditions leads to a spectral distribution of energy and the successive passage to the energy spectral density is justified only for L→∞. In the case of box of finite size, even if the spectral distribution obtained by imposing the boundary conditions is not dense and the relative frequency spacing between the spectral lines with lower frequency is wide, it happens that the spectral density obtained after the passage from discrete spectrum to dense spectrum fits the experimental evidence, and this is the only reason that, to our opinion, can be invoked to support the approach based on the boundary conditions and on the discrete spectrum.

Exactly as it happens with Bose’s derivation, the use of the triple integral (16) obscures the role of the multiplicity of energy eigenvalues, because the continuous variables (x,y,z) lead, through the successive change of continuous variables, to the continuous frequency, skipping at all the discrete frequency and, with it, the multiplicity of energy eigenvalues and the distribution of energy and entropy in the domain of the discrete frequency.

To further substantiate our claim of novelty, we point out that the density of states of massive particles is derived in many papers from the multiplicity of energy eigenvalues but the density of states of photons differs from the density of states of massive particles because, as it is apparent from (6) and as already discussed in one of the previous sections, the continuous version of the discrete energy of the photon, hence the continuous version of its discrete frequency, is proportional to $\sqrt{s}$, while the energy of the massive particle is proportional to *s*. To our best knowledge, the passage from the discrete index *n* to the continuous frequency ν through (3) and through the successive change of variables (6) is proposed here for the first time. This passage allows us to derive energy spectral density and entropy spectral density from energy distribution and entropy distribution in the domain of the eigenfrequencies, hence from the multiplicity of energy eigenvalues.

## 5. Comparison with the Poisson Distribution of the Occupancy Numbers

The photonic occupancy number of the coherent state follows the Poisson distribution. One could figure out a collection of independent coherent states at the frequencies $\left\{\nu_{n}\right\}$. If the expected occupancy number is small at each one of the frequencies, then it is reasonable to expect that the resulting radiation is virtually non-coherent and that the statistics of the photonic occupancy numbers approaches that of the thermal state.

In the following we compare entropies of different probability distributions with the same energy spectral distribution. To this aim, we take for the probability $P_{i,j,k}$ that one photon occupies state (i,j,k)

(19) $$\begin{array}{c} P_{i,j,k}=\frac{\mu_{r_{i,j,k}}}{\mu_{R}},\;\forall \left(i,j,k\right)\in M_{n}, \end{array}$$

where

$$\mu_{R}=\sum_{i,j,k}\mu_{r_{i,j,k}}.$$

The expected energy of the *n*-th eigenfrequency is

$$\sum_{\left(i,j,k\right)\in M_{n}}\mu_{R}h\nu_{i,j,k}P_{i,j,k}=\mu_{r_{n}}m_{n}h\nu_{n},$$

that is the one already introduced in Equation (10).

It is worth observing that the Poisson distribution, besides being the distribution that characterizes the coherent state, is also in strict connection with the multinomial distribution, recently proposed in [13] for the occupancy numbers of the canonical state of the ideal gas. According to [13], the joint probability distribution of the occupancy numbers in the canonical ensemble is multinomial:

$$\begin{array}{c} P_{\bar{r}}=R!\prod_{i,j,k}\frac{P_{i,j,k}^{r_{i,j,k}}}{r_{i,j,k}!}, \end{array}$$

where *R* is the total number of photons,

(20) $$\begin{array}{c} \sum_{i,j,k}r_{i,j,k}=R, \end{array}$$

which, in the canonical ensemble approach, is assumed to be fixed and known. However, in the case of photons inside a cavity, *R* is random, so we take a weighted average of the multinomial distributions with weights equal to the distribution of *N*:

(21) $$\begin{array}{c} P_{\bar{r}}=P_{R}R!\prod_{i,j,k}\frac{P_{i,j,k}^{r_{i,j,k}}}{r_{i,j,k}!}, \end{array}$$

where, again, (20) is understood. The actual probability distribution of *R* is that of the random variable obtained from the sum of geometrically distributed random variables, see [14]. Hower, this distribution is untractable. Assuming that the expectation of *R* is large, say, greater than 30, and since *R* is the sum of a large number of discrete random variables, we can approximate its distribution to a Poisson distribution with expected value $\mu_{R}$ of the Poissonian random variable, see also [15]

$$P_{R}=\frac{e^{-\mu_{R}}\mu_{R}^{R}}{R!}.$$

Inserting the above distribution and (20) in (21), we see that the distribution of the occupancy numbers is the product of Poisson distributions:

(22) $$\begin{array}{cc} P_{\bar{r}} & =e^{-\mu_{R}}\mu_{R}^{R}\prod_{i,j,k}\frac{P_{i,j,k}^{r_{i,j,k}}}{r_{i,j,k}!}\\ \; & =\prod_{i,j,k}\frac{e^{-P_{i,j,k}\mu_{R}}{\left(P_{i,j,k}\mu_{R}\right)}^{r_{i,j,k}}}{r_{i,j,k}!}\\ \; & =\prod_{i,j,k}\frac{e^{-\mu_{r_{i,j,k}}}{\left(\mu_{r_{i,j,k}}\right)}^{r_{i,j,k}}}{r_{i,j,k}!}, \end{array}$$

where, in the last equality, we substitute (19). In practice, the product of geometric distributions that characterize the thermal state becomes here the product of Poisson distributions with the same mean values.

The entropy of the individual Poisson distribution with parameter $\mu_{r}$ is equal to

(23) $$\begin{array}{cc} S_{r} & =\mu_{r}\left(1-\log\left(\mu_{r}\right)\right)+E\left\{\log\left(r!\right)\right\}, \end{array}$$

where E{·} is the expectation over the Poisson distribution of the function inside the curly brackets:

(24) $$\begin{array}{c} E\left\{\log\left(r!\right)\right\}=\sum_{r=0}^{\infty }\frac{e^{-\mu_{r}}\mu_{r}^{r}\log\left(r!\right)}{r!}. \end{array}$$

**Figure 2:**
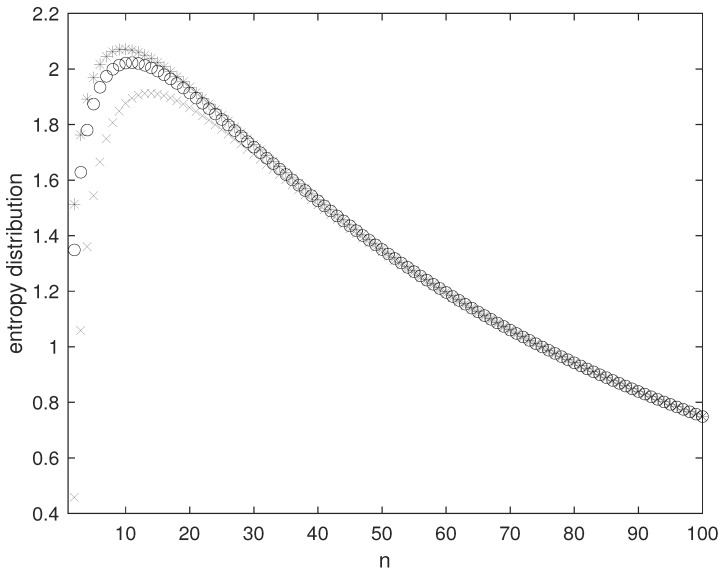
Entropy distribution $S_{r_{n}}$ obtained using the smooth multiplicity $g_{n}=\pi \sqrt{n}/4$. Temperature 300 Kelvin, side of the box $6\cdot 10^{-5}$ meters. Asterisks: geometric distribution. Circles: Poisson distribution. Crosses: rightmost term of (25).

When the expected number of photons in a quantum state tends to zero, the expectation in (23) tends to zero, leading to

(25) $$\begin{array}{cc} S_{r,geo}\geq S_{r,poi} & \geq \mu_{r}\left(1-\log\left(\mu_{r}\right)\right), \end{array}$$

where the first inequality is consequence of the maximum entropy property of the geometric distribution, while the rightmost term is obtained by neglecting the non-negative expectation in the entropy of the Poisson distribution (23) and is equal to the right-hand side of (9). Figure 2 reports the entropy distribution in the domain of the eigenfrequency calculated with the smooth discrete multiplicity $\pi \sqrt{n}/4$ for the geometric distribution, the Poisson distribution, and for the rightmost term of (25).

Using again the density of states and the rightmost term of (25), and using

$$\int_{x=0}^{\infty }\frac{x^{2}}{e^{x}-1}dx=2\zeta \left(3\right)\approx 2.4,\;\;\;\int_{x=0}^{\infty }\frac{x^{2}\log\left(e^{x}-1\right)}{e^{x}-1}dx\approx 5.95,$$

for the total entropy we obtain

$$\begin{array}{cc} S_{\bar{r}} & \approx \int_{\nu =0}^{\infty }\frac{4\pi L^{3}\nu^{2}}{c^{3}}(\mu \left(\nu \right)\left(1-\log\left(\mu \left(\nu \right)\right)\right)d\nu \\ \; & =\frac{4\pi L^{3}}{c^{3}\beta^{3}h^{3}}\left(2\zeta \left(3\right)+5.95\right)=105\cdot \frac{L^{3}}{c^{3}\beta^{3}h^{3}}, \end{array}$$

which is remarkably close to (13).

## 6. Conclusions

In the paper we have reviewed the past derivations of Bose’s entropy spectral density and presented a new one that is explicitly based on the multiplicity of the energy eigenvalues resulting from the imposition of the boundary conditions on the spatial boundaries of the blackbody. Among the approached mentioned in the paper, the author’s favor goes to the one of Feynman, which seems to better reproduce the physics of the blackbody. This approach, discussed by Feynman only for energy, not for entropy, is not based on the eigenstates of the photons inside the box and leads directly to the energy spectral density, without any passage from the discrete domain of the eigenfrequencies to the domain of the continuous frequency. The derivation of the entropy spectral density from Feynman’s approach is left to future investigation.

## Data Availability

No new data were created or analyzed in this study. Data sharing is not applicable to this article.
